# Pro-Inflammatory Microglia Exacerbate High-Altitude-Induced Cognitive Impairment by Driving Lipid Droplet Accumulation in Astrocytes

**DOI:** 10.3390/antiox14080918

**Published:** 2025-07-26

**Authors:** Xiaoyang Fan, Sitong Cao, Yujie Fang, Li Zhu, Xueting Wang

**Affiliations:** Institute of Special Environmental Medicine, Co-Innovation Center of Neuroregeneration, Nantong University, Nantong City 226009, China; 2325310001@stmail.ntu.edu.cn (X.F.); cst229323@stimail.ntu.edu.cn (S.C.); 2425310012@stmail.ntu.edu.cn (Y.F.)

**Keywords:** high-altitude cognitive impairment, lipid droplets, microglia, astrocyte, minocycline, IL-1β, inflammation

## Abstract

High-altitude cognitive impairment (HACI) results from acute or chronic exposure to hypoxic conditions. Brain lipid homeostasis is crucial for cognitive function, and lipid droplet (LD) accumulation in glia cells is linked to cognitive decline in aging and stroke. However, whether high-altitude exposure affects brain lipid homeostasis is unclear. Microglia, key regulators of brain homeostasis and inflammation, play a significant role in pathological cognitive impairment and are implicated in LD formation. This study investigates whether lipid dysregulation contributes to HACI and explores microglia-driven mechanisms and potential interventions. Mice were exposed to a simulated 7000 m altitude for 48 h, followed by a week of recovery. Cognitive function and LD accumulation in brain cells were assessed. Microglia were depleted using PLX5622, and mice were exposed to hypoxia or lipopolysaccharide (LPS) to validate microglia’s role in driving astrocytic LD accumulation and cognitive decline. Minocycline was used to inhibit inflammation. In vitro, co-culture systems of microglia and astrocytes were employed to confirm microglia-derived pro-inflammatory factors’ role in astrocytic LD accumulation. Hypobaric hypoxia exposure induced persistent cognitive impairment and LD accumulation in hippocampal astrocytes and microglia. Microglia depletion alleviated cognitive deficits and reduced astrocytic LD accumulation. Hypoxia or LPS did not directly cause LD accumulation in astrocytes but activated microglia to release IL-1β, inducing astrocytic LD accumulation. Microglia depletion also mitigated LPS-induced cognitive impairment and astrocytic LD accumulation. Minocycline reduced hypoxia-induced LD accumulation in co-cultured astrocytes and improved cognitive function. Hypoxia triggers pro-inflammatory microglial activation, leading to LD accumulation and the release of IL-1β, which drives astrocytic LD accumulation and neuroinflammation, exacerbating HACI. Minocycline effectively restores brain lipid homeostasis and mitigates cognitive impairment. This study provides novel insights into HACI mechanisms and suggests potential therapeutic strategies.

## 1. Introduction

Rapid ascent to high altitudes or prolonged stays at high altitudes can induce high-altitude cognitive impairment (HACI) [1]. This condition is prevalent and primarily manifests as declined learning and memory capacity, impaired decision-making ability, and reduced work efficiency, with its cognitive dysfunction often being irreversible [2,3]. Research indicates that the severity of cognitive impairment is significantly correlated with altitude and exposure duration [4]. Although HACI is widely recognized, its precise pathogenesis remains unclear, and existing conventional drugs for cognitive disorders lack specificity [5]. Given the non-regenerative nature of the nervous system, preventing HACI holds far greater importance than post-onset therapeutic interventions. Therefore, elucidating the pathogenic mechanisms of HACI is critical for developing effective preventive strategies. The cognitive impairment induced by high-altitude environments is undoubtedly linked to hypoxia; as the most oxygen-consuming organ, the brain exhibits exceptional sensitivity to oxygen deprivation [6]. Animal studies demonstrate that high-altitude exposure triggers oxidative stress in mouse neurons due to hypoxia, accompanied by aberrant activation of astrocytes and microglia, leading to markedly elevated neuroinflammation [7,8,9]. While some anti-inflammatory drugs have shown preventive potential against high-altitude brain injury [10,11], they fail to reverse established neurological damage, suggesting the need to explore novel mechanisms underlying high-altitude brain disorders, particularly HACI.

The brain is one of the most lipid-rich organs in the human body, with lipids accounting for approximately 60% of its total weight [12]. Under physiological conditions, various lipid components in the brain maintain a dynamic equilibrium through synthesis, degradation, transport, and metabolism, a precisely regulated balance termed lipid homeostasis [13]. Recent studies have revealed that lipid homeostasis plays a pivotal role in maintaining normal cognitive function, and its dysregulation is closely associated with various cognitive impairments, including stroke [14], Alzheimer’s disease (AD) [15], and neonatal hypoxic-ischemic encephalopathy [16]. Notably, the high-altitude hypoxic environment can significantly alter lipid metabolism characteristics: under hypoxia, the body enhances fatty acid oxidation to meet energy demands, leading to markedly increased lipid metabolic activity and efficiency [17]. Research indicates that high-altitude-adapted individuals exhibit adipose tissue with higher metabolic activity, enabling more effective regulation of lipid storage and mobilization [18,19]. Conversely, individuals with poor lipid metabolic adaptation are prone to high-altitude acclimatization disorders and related diseases [20]. Based on this evidence, we propose a scientific hypothesis that the pathogenesis of HACI, a common high-altitude cerebral disorder, may be potentially associated with lipid metabolism dysregulation.

Due to the presence of the blood–brain barrier, cerebral lipid metabolism exhibits unique regulatory characteristics. Astrocytes, as the primary site of lipid synthesis in the central nervous system (CNS), produce various essential lipid molecules, including phospholipids, fatty acids, and cholesterol, providing both energetic and structural support for neurons [21]. The maintenance of neuronal function critically depends on lipid uptake from glial cells [22]. Insufficient lipid uptake impairs synaptic formation [23], while excessive lipid accumulation disrupts synaptic vesicle recycling and neurotransmitter release [24]. Under conditions of lipid metabolic imbalance (referring to dysregulation of neural lipid homeostasis involving cholesterol, phospholipids, and sphingolipids), astrocytes store excess lipids in the form of lipid droplets (LDs), a process accompanied by increased secretion of inflammatory factors [25,26,27]. Moreover, abnormal LD accumulation triggers neuroinflammatory responses, promoting excessive microglial activation and leading to aberrant synaptic phagocytosis [28]. Notably, under excitotoxic or other stress conditions, astrocytes may exert neuroprotective effects by sequestering polyunsaturated fatty acids and their peroxidation products within lipid droplets (LDs) [29]. This suggests that LD formation exhibits context-dependent dual effects.

Previous studies have demonstrated that microglia play a pivotal driving role in the pathogenesis of high-altitude brain disorders under hypoxic conditions. As the predominant immune regulators in the CNS, microglia are indispensable for maintaining cerebral immune homeostasis [30]. Our preliminary experiments revealed that high-altitude exposure induces significant amnesic behaviors in mice, which are closely associated with microglia-mediated neuroinflammation and excessive synaptic phagocytosis [31]. Further investigations showed that hypoxic conditions at high altitudes trigger pathological swelling of astrocytes in a microglia-dependent manner [32]. Nevertheless, it remains unclear whether microglia contribute to disease progression by disrupting lipid metabolic homeostasis during HACI development. To definitively establish their causative role in hypoxia-induced neuropathology, we employed pharmacological microglial depletion using PLX5622, a brain-penetrant and highly selective colony-stimulating factor 1 receptor (CSF1R) inhibitor. This approach capitalizes on the fundamental dependence of microglial survival on CSF1R signaling. PLX5622 achieves > 90% microglial depletion by competitively inhibiting CSF1R phosphorylation, as rigorously validated in a prior study [33]. This selective ablation strategy enables unambiguous determination of microglia’s contributions to HACI pathogenesis. Therefore, this study aims to investigate the regulatory role of lipid homeostasis imbalance in HACI pathogenesis and to elucidate the molecular mechanisms by which glial cells (particularly microglia) modulate hypoxia-induced lipid metabolic dysregulation.

## 2. Materials and Methods

### 2.1. Animals and Treatments

The C57BL/6J mice used in this study were provided by the Experimental Animal Center of Nantong University. The mice were housed in a specific pathogen-free environment with controlled temperature (23 ± 2 °C), humidity (45–60%), and a 12 h light/dark cycle, with ad libitum access to food and water. All experimental procedures were performed in strict compliance with the guidelines approved by the Animal Care and Use Ethics Committee of Nantong University (approval ID: S20240425-003) and the Jiangsu Province Animal Care Ethics Committee (approval ID: SYXK[SU]2007-0021).

Hypobaric hypoxia (HH) exposure. Here, 8-week-old male mice were placed in an HH chamber (ProOx-830, TOW) or same-sized normobaric normoxia (NN) chamber. The pressure of HH chamber was reduced at a rate of 5 m/s to simulate an altitude of 7000 m, maintained for 48 h, and then returned to sea level at the same rate (5 m/s).

Minocycline rescue experiment. Here, 8-week-old male mice received intraperitoneal (i.p.) injections of minocycline (30 mg/kg/day) once daily for 3 consecutive days, followed by HH exposure. Vehicle control mice received an equivalent volume of saline.

Lipopolysaccharide (LPS)-induced systemic inflammation model. Here, 8-week-old male mice were injected intraperitoneally with LPS (L2630, Sigma, St. Louis, MO, USA) at 1 mg/kg/day once daily for 4 consecutive days [34]. Vehicle control mice received an equivalent volume of saline.

PLX5622 (microglial depletion) treatment. Here, 6-week-old male mice were fed a diet containing 1200 ppm PLX5622 (D20010801, SYSE, Suzhou, China) or control chow for 14 days. On Day 13, mice underwent HH exposure (or LPS injection on Day 11), with ad libitum access to food.

### 2.2. Morris Water Maze (MWM) Assay

The MWM assay followed our established protocol [35]. A circular pool (150 cm diameter) was divided into four quadrants (NW, NE, SE, SW) with visual cues placed around each edge. The pool contained opaque water (TiO_2_ suspension) maintained at 21–22 °C. During training (Days 1–5), a visible platform was first placed in the SW quadrant (Day 1), and mice were introduced from the other three quadrants. If a mouse failed to locate the platform within 60 s, it was guided to the platform and allowed to remain for 20 s. The platform was then rotated to the NW, NE, and SE quadrants for subsequent trials. From Days 2 to 5, a hidden platform (SW quadrant) was used, and the first latency to reach it within 60 s was recorded from three entry points. On Day 6 (probe trial), the platform was removed, and mice entered from the EN quadrant. We recorded the escape latency to cross the original platform location, the number of crossings within 60 s, swimming trajectory, and average speed.

To study HH effects on spatial learning/memory, mice underwent HH exposure before training and probe trials. To evaluate minocycline’s rescue effect on HH-induced memory deficits, we used our prior method [31]: mice first completed standard training and probe trials, were grouped based on escape latency to ensure balanced baselines, and then underwent HH exposure. A probe trial was conducted immediately after HH exposure to assess memory retention.

### 2.3. Novel Object Recognition (NOR) Assay

The NOR assay was performed to evaluate cognitive changes in mice after HH or LPS treatment [36]. A 50 cm × 50 cm × 50 cm square arena contained two identical objects (A and B, 3 cm diameter) symmetrically placed. Mice were gently introduced facing the opposite wall, and exploration was defined as nose contact within 3 cm of an object. During the training phase, exploration time for both objects was recorded over 5 min. After a 12 h interval, object B was replaced with novel object C (similar size but distinct shape) for the test phase, and exploration time for objects A and C was measured. The discrimination index (%) = TC/(TA + TC) × 100% (TA: exploration time for object A; TC: exploration time for object C) was calculated to assess recognition memory.

### 2.4. Brain Tissue Lipid Content Measurement

Total cholesterol in brain tissue was measured using a commercial assay kit (Total Cholesterol Assay Kit, A111-1-1, Nanjing Jiancheng Bioengineering, Nanjing, China). Fresh brain tissue was homogenized in absolute ethanol, and the supernatant was collected after centrifugation. Following the manufacturer’s protocol, samples, standards, and reaction reagents were added to a 96-well plate and incubated at 37 °C for 30 min in the dark. Absorbance was measured at 500 nm using a microplate reader, and total cholesterol content was calculated based on the provided formula.

Free fatty acid content was analyzed using a Micro Free Aliphatic Acid Content Assay Kit (BC0595, Solarbio, Beijing, China). A standard curve was prepared (0.05–1 μmol/mL), and 0.1 g of fresh brain tissue was homogenized in isopropanol, followed by centrifugation to collect the supernatant. Reaction reagents were added according to the manufacturer’s instructions, and samples were incubated at room temperature for 15 min in the dark. Absorbance was measured at 550 nm, and free fatty acid levels were calculated based on the standard curve.

Triglyceride levels were determined using a Triglyceride Assay Kit (A110-1-1, Nanjing Jiancheng Bioengineering, Nanjing, China) following the same procedure as total cholesterol measurement. The final triglyceride content was calculated based on the standard curve.

### 2.5. Cell Culture and Treatments

Astrocyte cultures were established from the cerebral tissues of 2-day-old C57BL/6J neonatal mice. Brain tissues were thoroughly dissociated with 0.05% trypsin to generate single-cell suspensions, which were then resuspended in DMEM/F-12 complete medium supplemented with 10% fetal bovine serum (10100147, Thermo, Waltham, MA, USA), 1% penicillin/streptomycin (SV30010, Hyclone, Logan, UT, USA), and 1% glutamine (35050061, Gibco, Grand Island, NY, USA). Cells were cultured at 37 °C under 5% CO_2_ until a mixed glial cell layer formed. After reaching confluence, floating cells were removed, and adherent cells were passaged using 0.25% trypsin. GFAP immunostaining confirmed that the purity of passaged astrocytes exceeded 99% [37].

Primary microglial cultures were prepared using the same single-cell suspension protocol as for astrocytes. The mixed glial cells were resuspended in DMEM/F-12 medium supplemented with 5 ng/mL GM-CSF (78017, STEMCELL, Vancouver, BC, Canada) and cultured at 37 °C with 5% CO_2_ for 14 days. Microglia floating in the culture supernatant were then collected and transferred to GM-CSF-free DMEM/F12 medium for resting culture for at least 24 h prior to experiments. Iba1 immunostaining confirmed that the purity of isolated microglia exceeded 98% [31,38].

For astrocyte–microglia Transwell co-culture, astrocytes were first seeded in 24-well plates for 24 h, followed by plating primary microglia (1 × 10^6^ cells/mL) in the Transwell inserts (0.4 μm pore size) to establish a non-contact co-culture system, allowing paracrine communication through shared medium. For direct co-culture, astrocytes were seeded in confocal dishes until reaching ~40% confluency, and then primary microglia (5 × 10^5^ cells/mL) were directly added to create a cell contact co-culture model.

For hypoxia treatment, cells were cultured in a hypoxia workstation (Invivo2400, Ruskinn, UK) under 1% O_2_ and 5% CO_2_ for 24 h. For minocycline treatment, cells were pre-incubated with 10 μM minocycline (HY-17412, MCE, New Jersey, USA) before hypoxia exposure. For LPS+Nigericin (Nig) treatment, cells were first treated with 10 ng/mL LPS (prepared in serum-free DMEM/F-12) for 3.5 h followed by 10 μM Nig (28643-80-3, Sigma, St. Louis, MO, USA) for 40 min [39]. To prepare conditioned media, primary microglia culture media from normoxia or hypoxia treatment were collected and mixed with fresh medium at 1:2 ratio, designated as normoxia-conditioned medium (NCM) and hypoxia-conditioned medium (HCM), respectively. Primary astrocytes were then cultured with NCM or HCM under hypoxia for 24 h. For IL-1β treatment, astrocytes were incubated with 10 ng/mL IL-1β (HY-P7073A, MCE) in DMEM/F12 medium for 6 h [40].

### 2.6. LD Staining

Fixed glial cells or tissue samples (4% paraformaldehyde) were subjected to immunofluorescence labeling, followed by incubation with 1 μg/mL BODIPY 493 (GC42959, GLPBIO, Montclair, CA, USA) for 15 min at room temperature in the dark. Imaging was performed using a Zeiss Axio Observer 7 microscope (Zeiss, Jena, Germany) equipped with Apotome III structured illumination module under a FITC filter.

After fixation and immunofluorescence labeling, astrocytes were incubated with 1 μM Nile Red (GC15539, GLPBIO) for 30 min at room temperature in the dark. Stained samples were imaged using a Zeiss Axio Observer 7 microscope equipped with Apotome III structured illumination module under a Cy3 filter.

### 2.7. Immunofluorescence Staining

Brain tissues fixed with 4% paraformaldehyde were sectioned at 40 μm thickness and permeabilized with 0.03% Triton X-100, while cultured cells were directly fixed. After blocking with 10% bovine serum albumin, samples were incubated overnight at 4 °C with primary antibodies (Anti-GFAP, 3610, Cell Signaling Technology; Anti-Iba1, OB-PGP049, Osaisbiofarm, Huzhou, China; Anti-PLIN2, HY-P83553, MCE; Anti-PSD95, 20665-1-AP, Proteintech, Wuhan, China; Anti-TUJ1, 66375-1-1g, Proteintech), followed by corresponding fluorescent secondary antibodies (Goat anti-Mouse Cy3, 115-165-003; Donkey anti-Guinea Pig Cy3, 706-165-148; Donkey anti-Rabbit Alexa 488, 711-545-152; Goat anti-Rabbit Cy3, 111-165-003; Donkey anti-Guinea Pig Alexa 488, 706-545-148; Donkey anti-Mouse Alexa 647, 715-605-151, all from Jackson ImmunoResearch, West Grove, PA, USA) for 2 h at room temperature in the dark. Nuclei were counterstained with DAPI (D1306, Thermo, Waltham, MA, USA) for 5 min, and images were acquired using a Zeiss Axio Observer 7 microscope with Apotome III microscope. Fluorescence intensity quantification and colocalization analysis were performed using Fiji ImageJ 1.51w software (https://imagej.net/software/fiji/downloads (accessed on 1 March 2020)). The circularity coefficient of microglia in brain tissue sections was quantitatively analyzed. Briefly, 8-bit grayscale images were processed in Fiji ImageJ: the Threshold module was applied to set binary thresholds, and individual microglia were manually selected using the Wand Tool. Under Set Measurements, the Shape descriptors option was enabled, and the Measure function was executed to obtain morphological parameters, with Circularity (Circ) representing the roundness of microglia.

### 2.8. RNA Extraction and Quantitative PCR (q-PCR)

Total RNA was extracted from fresh brain tissue or cultured cells using the Trizol method, and cDNA was synthesized by reverse transcription using the HiScript IV All-in-One Ultra RT SuperMix for qPCR kit (R433-01, Vazyme, Nanjng, China). q-PCR was then performed using the AceQ qPCR SYBR Green Master Mix (High ROX Premixed) kit (Q141-02, Vazyme). The primer sequences were as follows:

*Srebf1* (NM_001313979.1) Forward: 5′-CGACTACATCCGCTTCTTGCAG-3′, Reverse: 5′-CCTCCATAGACACATCTGTGCC-3′; *Tnfa* (NM_001278601.1) Forward: 5′-AAGCCTGTAGCCCACGTCGTA-3′, Reverse:5′-GGCACCACTAGTTGGTTGTCTTTG-3′; *Il1b* (XM_006498795.5) Forward: 5′-TGCCACCTTTTGACAGTGATG-3′, Reverse: 5′-TGATGTGCTGCTGCGAGATT-3′; *Actb* (NM_007393.5) Forward: 5′-CATCCGTAAAGACCTCTATGCCAAC-3′, Reverse: 5′-ATGGAGCCACCGATCCACA-3′.

The q-PCR program consisted of three steps: denaturation at 95 °C for 10 s, annealing at 60 °C for 20 s, and extension at 72 °C for 20 s, for a total of 40 cycles. The relative gene levels were calculated using the ^ΔΔCt^ method.

### 2.9. Protein Extraction and Western Blotting

Microglia treated with LPS and Nig were lysed using ice-cold RIPA buffer (BL504A, Biosharp, Nanjing, China) to extract total proteins. Protein concentration was measured and normalized using the Bicinchoninic Acid Assay (BCA) kit (Q141-02, Vazyme). The proteins were then separated by SDS-PAGE and transferred onto PVDF membranes. Then, membranes were incubated with anti-IL-1β (ab9722, Abcam, Cambridge, UK) and anti-β-actin (66009-1-lg, Proteintech, Wuhan, China) antibodies, followed with incubation of HRP-conjugated secondary antibodies (Goat anti-Mouse, 115-035-003, Jackson ImmunoResearch, West Grove, PA, USA) to visualize the target proteins via chemiluminescence. Finally, band intensity was quantified using Fiji ImageJ software to determine relative protein levels.

### 2.10. Statistical Analysis

The data were presented as mean ± SEM. GraphPad Prism v8.0 was used for statistical analysis, including Student’s *t*-test (for two-group comparisons) and two-way ANOVA (for multi-group comparisons) followed by Dunnett’s post hoc test. Significance levels were defined as * *p* < 0.05, ** *p* < 0.01, and *** *p* < 0.001, and n.s. shows no significant difference.

## 3. Results

### 3.1. Sustained Cognitive Dysfunction and Glial Activation in Mice a Week Post-Recovery from Acute HH Exposure

**Figure 1:**
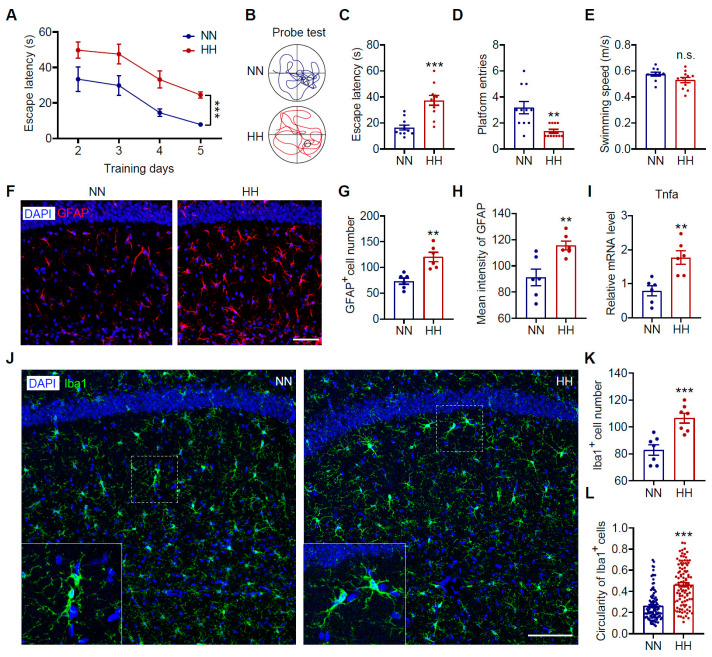
HH exposure impaired learning and memory function in mice, accompanied by microglial and astrocytic activation in the brain. Eight-week-old C57BL/6J mice were exposed to simulated high-altitude conditions (7000 m, 48 h) and subsequently subjected to the MWM test. (**A**) Escape latency of mice to locate the platform during training days 2–5 (*n* = 11). (**B**–**E**) Probe test performance, including the movement trajectory of mice (**B**), escape latency to reach the target platform area (**C**), number of crossings over the platform area (**D**), and average swimming speed (**E**) (*n* = 11). (**F**) Representative images of mouse brain sections immunolabeled with anti-GFAP antibody (red) and counterstained with DAPI (blue). Scale bar = 50 μm. (**G**) Quantification of GFAP^+^ cells in the CA1 region (*n* = 6). (**H**) Average fluorescence intensity of GFAP in the CA1 region (*n* = 6). (**I**) q-PCR analysis of Tnfa mRNA levels in the CA1 region of mouse brains (*n* = 6). (**J**) Representative images of mouse brain sections immunolabeled with anti-Iba1 antibody (Green) and counterstained with DAPI (blue). Scale bar = 100 μm. (**K**) Quantification of Iba1^+^ cells in the CA1 region (*n* = 7). (**L**) Circularity coefficient of Iba1^+^ cells (20 cells per mouse from 5 mice per group). Data were analyzed using two-way ANOVA followed by Sidak’s multiple comparisons test (**A**) or Student’s *t*-test (**C**–**E**,**G**–**I**,**K**,**L**). ** *p* < 0.01, *** *p* < 0.001; n.s., not significant.

Our previous studies confirmed that acute HH exposure can induce significant amnesia in mice [31]. However, whether the cognitive impairment caused by HH exposure is persistent remains unclear. In this study, we employed a simulated 7000 m altitude HH exposure model lasting 48 h to systematically evaluate the spatial learning and memory abilities of mice using the MWM test. During the 5-day training period, the escape latency of the HH group was consistently significantly longer than that of the NN group (Figure 1A, *p* < 0.001). In the probe period, the HH group exhibited reduced trajectory time in the target quadrant (Figure 1B), significantly prolonged latency to first reach the target quadrant (Figure 1C, *p* < 0.001), and significantly fewer platform crossings (Figure 1D, *p* < 0.01), with no statistical difference in average swimming speed between the two groups (Figure 1E, *p* > 0.05). These results demonstrate that the cognitive impairment induced by HH exposure persists after returning to plain conditions.

Abnormal activation of glial cells and related neuroinflammatory responses may be critical pathological mechanisms mediating cognitive dysfunction after HH exposure [31,41]. However, whether these pathological processes persist after returning to plain conditions remains unknown. We further examined glial cell activation in the CA1 region of the mouse brain one week after returning to NN conditions. Compared to the NN group, the HH group showed a significant increase in GFAP^+^ cell number (Figure 1F, G, *p* < 0.01) and markedly enhanced intensity (Figure 1H, *p* < 0.01) in the CA1 region. Additionally, the number of Iba1^+^ cells was significantly higher (Figure 1J-K, *p* < 0.001), and the morphological parameter (circularity) of microglia was significantly increased (Figure 1L, *p* < 0.001). Furthermore, TNFα expression levels in the hippocampal tissue of the HH group were significantly upregulated (Figure 1I, *p* < 0.01). These results indicate that HH exposure-induced glial cell activation and neuroinflammatory responses persist even after returning to a normoxic environment. Therefore, acute HH exposure not only leads to significant spatial learning and memory impairment but also induces sustained glial cell activation and neuroinflammatory responses after returning to normoxic conditions, which may serve as a critical pathological basis for long-term cognitive dysfunction.

### 3.2. HH Exposure Induces LD Accumulation in Microglia and Astrocytes in the Mouse Brain

**Figure 2:**
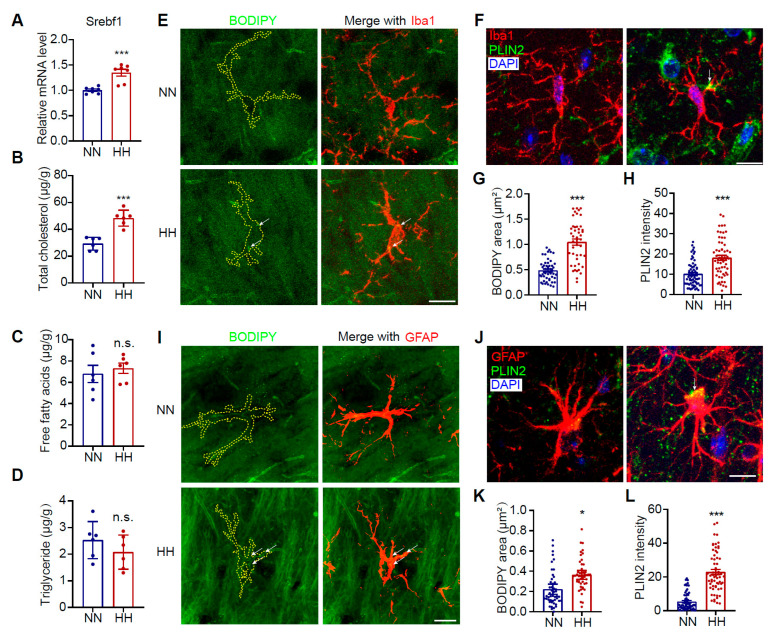
HH exposure induces increased lipid synthesis in microglia and astrocytes in the CA1 region of the mouse brain. C57BL/6J mice were exposed to simulated high-altitude conditions (7000 m, 48 h). (**A**) qRT-PCR analysis of the expression of lipid synthesis-related molecule Srebf1 in the CA1 region (*n* = 7). (**B**–**D**) Total cholesterol, free fatty acids and triglyceride content in mouse brains (*n* = 6). (**E**) Representative images of mouse brain sections immunolabeled with anti-Iba1 antibody (red) and co-stained with the LD dye BODIPY 493 (green). (**F**) Representative images of mouse brain sections immunolabeled with anti-Iba1 (red) and anti-PLIN2 (green)antibodies and counterstained with DAPI (blue). (**G**) Quantitative analysis of the BODIPY 493 fluorescence area in Iba1^+^ cells in the CA1 region (*n* = 50; 10 cells per mouse from 5 mice per group). (**H**) Quantitative analysis of PLIN2 fluorescence intensity in Iba1^+^ cells in the CA1 region (*n* = 50; 10 cells per mouse from 5 mice per group). (**I**) Representative images of mouse brain sections immunolabeled with anti-GFAP (red) antibody and co-stained with BODIPY 493 (green). (**J**) Representative images of mouse brain sections immunolabeled with anti-GFAP (red)and anti-PLIN2 (green)antibodies and counterstained with DAPI (blue). (**K**) Quantitative analysis of the BODIPY 493 fluorescence area in GFAP^+^ cells in the CA1 region (*n* = 50; 10 cells per mouse from 5 mice per group). (**L**) Quantitative analysis of PLIN2 fluorescence intensity in GFAP^+^ cells in the CA1 region (*n* = 50; 10 cells per mouse from 5 mice per group). Scale bar in fluorescence images = 10 μm. Data were analyzed using Student’s *t*-test. * *p* < 0.05, *** *p* < 0.001; n.s., not significant.

To investigate the impact of HH exposure on cerebral lipid metabolic homeostasis, we measured changes in lipid levels in brain tissues. Our findings revealed a significant upregulation in the expression of SREBP1, a key transcription factor for lipid synthesis (Figure 2A, *p* < 0.001). Cholesterol content in the hippocampal tissue was markedly increased (Figure 2B, *p* < 0.001), while no significant changes were observed in free fatty acids or triglyceride levels (Figure 2C,D). Given that glial cells are the primary regulators of lipid metabolism in the brain, we further quantified lipid accumulation in microglia and astrocytes. The results demonstrated a significant increase in neutral LD content within microglia of HH-exposed mice (Figure 2E,G, *p* < 0.001). Consistently, PLIN2, an LD surface marker protein, was also notably upregulated (Figure 2F,H, *p* < 0.001). This lipid accumulation aligns with the pro-inflammatory activation phenotype of microglia. As the predominant cell type responsible for lipid synthesis in the brain, astrocytes exhibited similar alterations, with both the neutral LD content (Figure 2I,K, *p* < 0.01) and PLIN2 level (Figure 2J,L, *p* < 0.001) significantly upregulated. Notably, no significant changes in neutral LDs or PLIN2 levels were detected in neurons following HH exposure (Figure S1). These findings suggest that HH exposure specifically induces enhanced lipid synthesis and LD accumulation in glial cells (including microglia and astrocytes). Such lipid metabolic dysregulation may serve as a key factor driving glial cell activation and exacerbating neuroinflammatory responses.

### 3.3. Microglia Depletion Attenuates HACI and Astrocytic LD Accumulation

**Figure 3:**
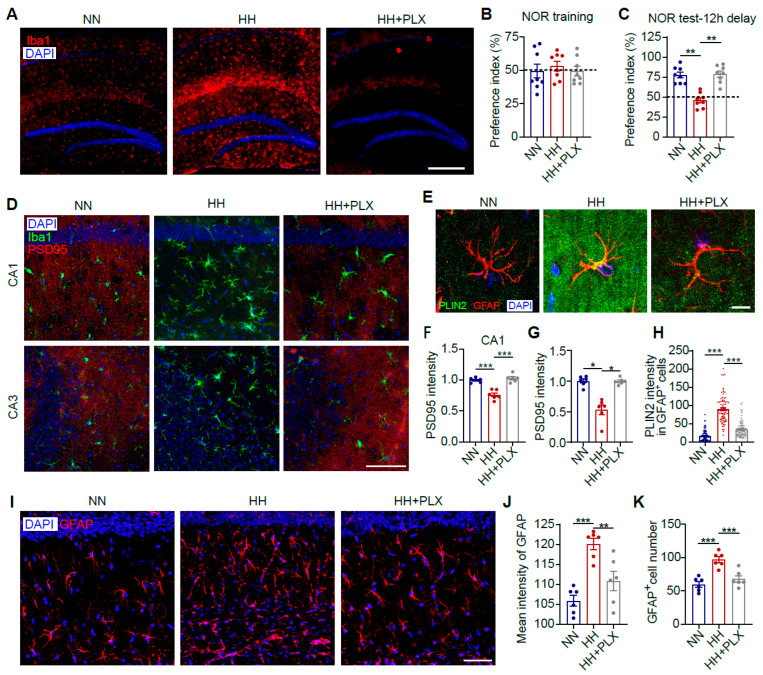
Microglia depletion alleviates HACI and reduces LD accumulation in astrocytes. C57BL/6J mice were fed PLX5622 for 14 days to deplete microglia and then exposed to a simulated high-altitude environment (7000 m, 48 h). (**A**) Representative images of mouse brain sections immunolabeled with anti-Iba1 (red) antibody and counterstained with DAPI (blue). Scale bar = 500 μm. (**B**) Preference index for object B during the training phase of the NOR test (*n* = 8). (**C**) Preference for object C during the testing phase of the NOR test (12 h delay, *n* = 8). (**D**) Representative images of brain sections immunolabeled with anti-Iba1 (green) and anti-PSD95 (red) antibodies and counterstained with DAPI (blue). Scale bar = 50 μm. (**E**) Representative images of mouse brain sections immunolabeled with anti-GFAP (red) and anti-PLIN2 (green) antibodies and counterstained with DAPI (blue). Scale bar = 10 μm. (**F**,**G**) Quantitative analysis of the average fluorescence intensity of PSD95 in the CA1 (**F**) and CA3 (**G**) regions (*n* = 6). (**H**) Quantitative analysis of PLIN2 signal intensity within GFAP^+^ cells in the CA1 region (*n* = 77; 15–16 cells per mouse from 5 mice per group). (**I**) Representative images of brain sections immunolabeled with anti-GFAP (red) antibody and counterstained with DAPI (blue). Scale bar = 50 μm. (**J**,**K**) Quantitative analysis of the average fluorescence intensity of GFAP (**J**) and the number of GFAP^+^ cells (**K**) in the CA1 region (*n* = 6). Data were analyzed using Student’s *t*-test. * *p* < 0.05, ** *p* < 0.01, *** *p* < 0.001. PLX, PLX5622.

Microglial activation is a prerequisite for astrocyte activation under hypoxic conditions [37,42]. To elucidate the mechanistic role of microglia in HH-induced cerebral lipid metabolic dysregulation, we established a microglia-specific depletion model using the CSF1R inhibitor PLX5622 (Figure 3A). NOR tests revealed that microglial depletion significantly ameliorated HH exposure-induced memory dysfunction in mice (Figure 3B,C, *p* < 0.01). Given that synaptic loss is a hallmark pathological feature of HACI [43], we further assessed changes in synaptic density. Microglial depletion completely abrogated HH-induced synaptic reduction (Figure 3D,F,G, *p* < 0.05), confirming that microglial activation is pivotal in mediating synaptic loss and HACI progression. To investigate the crosstalk between microglia and astrocytic lipid metabolism, we analyzed LD accumulation in CA1 astrocytes. The results demonstrated that microglial depletion markedly attenuated HH-triggered LD accumulation in astrocytes (Figure 3E,H, *p* < 0.001). Furthermore, microglial depletion suppressed astrocyte proliferation (Figure 3I,K, *p* < 0.001) and activation (Figure 3J, *p* < 0.05). These findings indicate that microglia directly contribute to HACI pathogenesis by driving synaptic loss, potentially through disrupting astrocytic lipid homeostasis and promoting their activation, thereby indirectly exacerbating cerebral lipid metabolic imbalance.

### 3.4. Hypoxia-Induced Astrocyte LD Accumulation Depends on Microglia-Derived Cytokines

**Figure 4:**
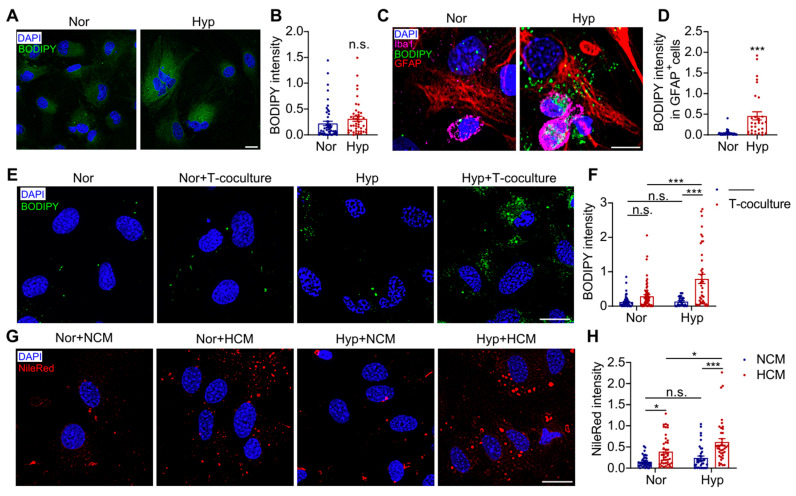
Cytokines secreted by hypoxic microglia exacerbate LD accumulation in astrocytes under hypoxic conditions. (**A**) Cultured astrocytes were cultured under 1% O_2_ for 24 h. Representative images showing BODIPY 493-labeled LDs (green) and DAPI-stained nuclei (blue). Scale bar = 20 μm. (**B**) Quantitative analysis of LD density in hypoxic astrocytes (*n* = 50). (**C**) Representative images of microglia-astrocyte co-culture system under 1% O_2_ for 24 h, immunostained with anti-Iba1 (pink) for microglia, anti-GFAP (red) for astrocytes, BODIPY 493 (green) for LDs, and DAPI (blue) for nuclei. Scale bar = 10 μm. (**D**) Quantitative analysis of LD density in co-cultured astrocytes (*n* = 30). (**E**) Representative images of Transwell co-culture system (upper chamber: microglia) under 1% O_2_ for 24 h, showing BODIPY 493-labeled LDs (green) and DAPI-labeled nuclei (blue) in astrocytes. Scale bar = 20 μm. (**F**) Quantitative analysis of LD density in astrocytes from Transwell system (*n* = 55). (**G**) Representative images of astrocytes treated with HCM or NCM for 24 h, stained with Nile Red for LDs (green) and DAPI (blue) for nuclei. Scale bar = 20 μm. (**H**) Quantitative analysis of LD density in astrocytes treated with different conditioned media (*n* = 40). Data were analyzed using Student’s *t*-test (**B**,**D**) or two-way ANOVA with Sidak’s multiple comparisons test (**F**,**H**). * *p* < 0.05, *** *p* < 0.001; n.s., not significant. Nor, normoxia; Hyp, hypoxia.

To further investigate the mechanism underlying hypoxia-induced LD accumulation in astrocytes, we conducted in vitro experiments. Intriguingly, cultured astrocytes exposed to hypoxia alone (1% O_2_, 24 h) showed no significant LD accumulation (Figure 4A,B). However, when co-cultured with microglia, hypoxia treatment markedly induced LD formation in astrocytes (Figure 4C,D, *p* < 0.001). To further verify whether microglial regulation of astrocytes is dependent on cell–cell contact, we employed a Transwell co-culture system. Remarkably, even under physical separation, hypoxia-treated microglia still significantly promoted astrocytic LD accumulation (Figure 4E,F, *p* < 0.001), suggesting this process is primarily mediated by soluble factors secreted from microglia. We further examined whether this microglia-derived cytokine-induced lipid accumulation in astrocytes depends on the hypoxic environment. Our data revealed that the microglial-conditioned medium (HCM) alone could induce astrocytic LD accumulation under normoxic conditions, while hypoxic exposure significantly potentiated this HCM-mediated effect (Figure 4G,H, *p* < 0.001). These findings demonstrate that astrocytic LD accumulation depends on extracellular signals secreted by hypoxia-activated microglia and that the hypoxic environment can synergistically enhance this process.

### 3.5. LPS-Activated Pro-Inflammatory Microglia Secrete IL-1β to Trigger Astrocyte LD Accumulation

**Figure 5:**
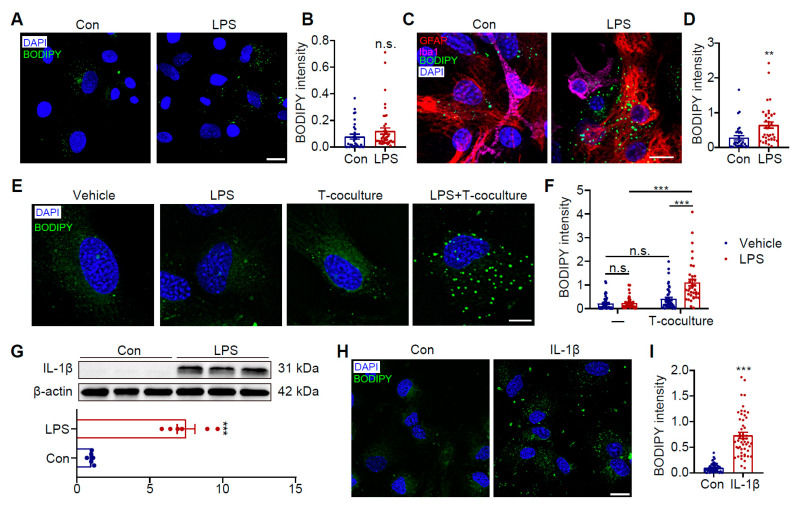
Activated microglia induce LD accumulation in astrocytes through the release of IL-1β. (**A**) Astrocytes treated with LPS+Nig, showing BODIPY 493-labeled LDs (green) and DAPI-stained nuclei (blue). Scale bar = 20 μm. (**B**) Quantitative analysis of LD density in LPS-treated astrocytes (*n* = 30). (**C**) Representative images of primary microglia-astrocyte co-culture system treated with LPS+Nig, immunostained with anti-Iba1 (pink), anti-GFAP (red), BODIPY 493 (green), and DAPI (blue). Scale bar = 20 μm. (**D**) Quantitative analysis of LD density in GFAP^+^ cells (*n* = 30). (**E**) Representative images of Transwell co-culture system (upper chamber: microglia) treated with LPS+Nig, showing BODIPY 493-labeled LDs (green) and DAPI-labeled nuclei (blue) in astrocytes. Scale bar = 10 μm. (**F**) Quantitative analysis of LD density in astrocytes from Transwell system (*n* = 40). (**G**) Western blot analysis of IL-1β in microglia treated with LPS+Nig (*n* = 6). (**H**) Representative images of IL-1β-treated astrocytes showing BODIPY 493-labeled LDs (green)and DAPI-stained nuclei (blue). Scale bar = 20 μm. (**I**) Quantitative analysis of LD density in IL-1β-treated astrocytes (*n* = 50). Data were analyzed using Student’s *t*-test (**B**,**D**,**G**,**I**) or two-way ANOVA with Sidak’s multiple comparisons test (**F**). ** *p* < 0.01, *** *p* < 0.001; n.s., not significant. Nor, normoxia; Hyp, hypoxia.

Studies have demonstrated that LPS can induce LD formation in various cell types, particularly macrophages [44,45]. We further investigated whether LPS could directly trigger LD accumulation in astrocytes. Notably, LPS combined with Nig treatment failed to directly induce LD formation in astrocytes (Figure 5A,B). However, when co-cultured with microglia, LPS+Nig treatment significantly promoted LD accumulation in astrocytes under both direct contact (Figure 5C,D, *p* < 0.01) and Transwell indirect co-culture conditions (Figure 5E,F, *p* < 0.001). These findings suggest that LPS+Nig activates microglia to release inflammatory factors, which subsequently induce astrocytic LD accumulation. Further analysis revealed that LPS+Nig treatment markedly increased production of the pro-inflammatory cytokine IL-1β in microglia (Figure 5G, *p* < 0.001). To confirm whether IL-1β is responsible for inducing LD accumulation in astrocytes, we treated astrocytes directly with recombinant IL-1β, which indeed significantly induced LD accumulation (Figure 5H,I, *p* < 0.001). These results conclusively demonstrate that microglia-derived IL-1β plays a pivotal role in mediating LD accumulation in astrocytes.

### 3.6. Microglia Drive Inflammation-Induced Astrocytic LD Accumulation and Cognitive Deficits

**Figure 6:**
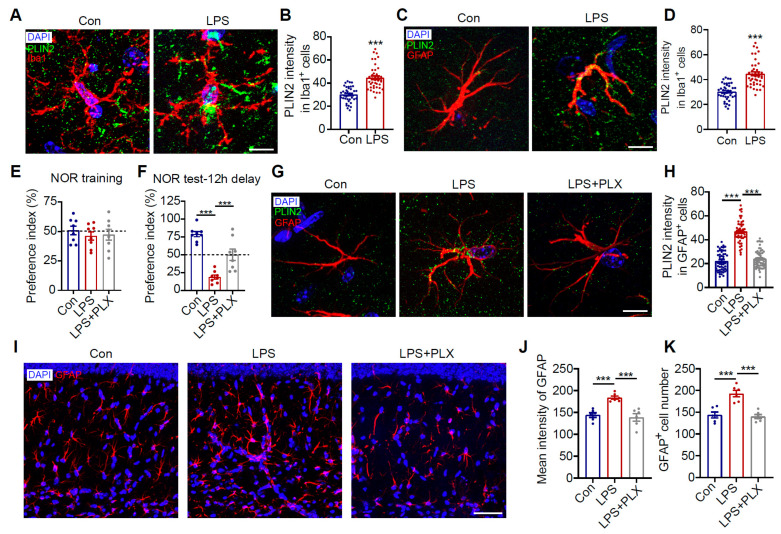
Microglia depletion attenuates LPS-induced astrocytic LD accumulation and cognitive impairment in mice. C57BL/6J mice were intraperitoneally injected with LPS. (**A**) Representative immunofluorescence images of the CA1 region stained with anti-PLIN2 (green) and anti-Iba1 (red) antibodies (DAPI, blue). Scale bar = 10 μm. (**B**) Quantification of PLIN2 fluorescence intensity within Iba1^+^ cells (*n* = 40; 8 cells per mouse from 5 mice per group). (**C**) Representative immunofluorescence images of the CA1 region stained with anti-PLIN2 (green) and anti-GFAP (red) antibodies (DAPI, blue). Scale bar = 10 μm. (**D**) Quantification of PLIN2 intensity within GFAP^+^ cells (*n* = 50; 10 cells per mouse from 5 mice per group). (**E**,**F**) Mice were fed PLX5622 to deplete microglia, followed by LPS injection. NOR test was performed to assess preference indices during the training phase and testing phase (12-h delay) (*n* = 8). (**G**) Representative immunofluorescence images of the CA1 region from PLX5622 + LPS-treated mice, stained with anti-PLIN2 (green) and anti-GFAP (red) antibodies (DAPI, blue). Scale bar = 10 μm. (**H**) Quantification of PLIN2 fluorescence intensity within GFAP^+^ cells (*n* = 52; 13 cells per mouse from 4s mice per group). (**I**) Representative immunofluorescence images of the CA1 region stained with anti-GFAP (red) antibody (DAPI, blue). Scale bar = 10 μm. (**J**,**K**) Quantification of average GFAP fluorescence intensity (**J**) and GFAP^+^ cell numbers (**K**) (*n* = 6). Data were analyzed using Student’s *t*-test. *** *p* < 0.001. PLX, PLX5622.

Our in vitro experiments confirmed that LPS significantly promotes LD accumulation in astrocytes within co-culture systems. Similar phenomena were observed in vivo. Intraperitoneal LPS injection successfully induced LD accumulation in both microglia and astrocytes in mouse brains (Figure 6A–D, *p* < 0.001) by triggering systemic inflammatory responses. Previous studies have reported that LPS induces cognitive dysfunction in mice [46]. We hypothesized that this process might be mediated by astrocytes through microglia. To test this hypothesis, we specifically depleted microglia using PLX5622. The results showed that microglial depletion not only significantly alleviated LPS-induced cognitive impairment (Figure 6E,F, *p* < 0.001) but also effectively suppressed LPS-triggered LD accumulation in hippocampal CA1 astrocytes (Figure 6G,H, *p* < 0.001) while markedly improving astrocyte activation status (Figure 6I–K, *p* < 0.001). These findings demonstrate that systemic inflammation activates microglia, which in turn induce LD accumulation and inflammatory changes in astrocytes through a cascade reaction, ultimately leading to cognitive dysfunction.

### 3.7. Minocycline Reduces Astrocytic LDs and Improves Cognitive Impairment in HH-Exposed Mice by Inhibiting Microglial Inflammation

**Figure 7:**
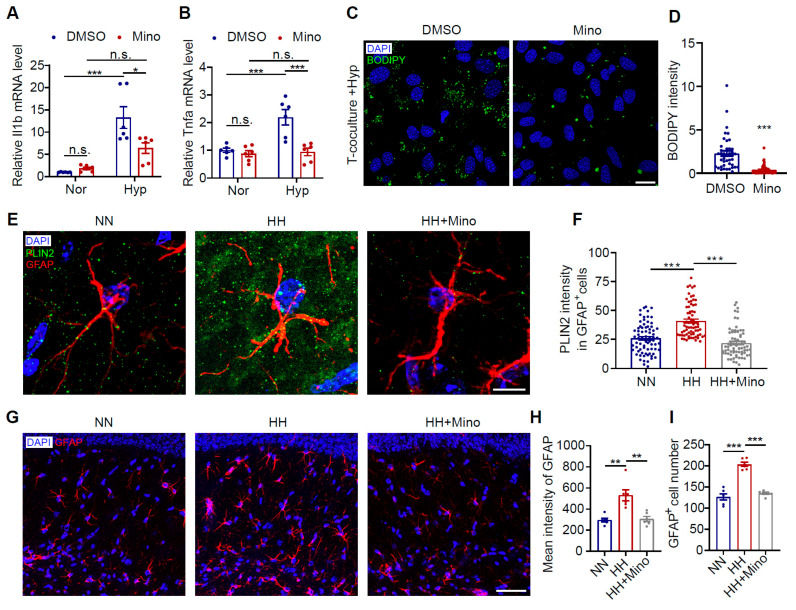
Minocycline reduces hypoxia-induced microglia-dependent astrocyte LD accumulation. (**A**,**B**) Primary microglia were treated with minocycline under hypoxic conditions. mRNA levels of pro-inflammatory cytokines Il1b and Tnfa were measured by qRT-PCR (*n* = 6). (**C**) Astrocytes and microglia were co-cultured in a Transwell system under hypoxia with minocycline treatment. LDs in astrocytes (lower chamber) were labeled with BODIPY 493 (green), and nuclei were counterstained with DAPI (blue). Scale bar = 20 μm. (**D**) Quantification of BODIPY intensity in astrocytes (*n* = 40). (**E**) Mice were intraperitoneally injected with minocycline followed by HH exposure. Brain sections of the CA1 region were immunostained with anti-PLIN2 (green) and anti-GFAP (red) antibodies (DAPI, blue). Scale bar = 10 μm. (**F**) Quantification of PLIN2 density within GFAP^+^ cells (*n* = 72; 9 cells per mouse from 8 mice per group). (**G**) Representative images of CA1 hippocampal region immunostained with anti-PLIN2 (red) antibody (DAPI, blue). Scale bar = 50 μm. (**H**,**I**) Quantification of GFAP mean fluorescence intensity (**H**) and GFAP^+^ cell numbers (**I**) in the CA1 region (*n* = 6). Data were analyzed using Student’s *t*-test (**D**,**F**,**H**,**I**) or two-way ANOVA with Sidak’s multiple comparisons test (**A**,**B**). * *p* < 0.05, ** *p* < 0.01, *** *p* < 0.001; n.s., no significant. Nor, normoxia; Hyp, hypoxia; Mino, minocycline. 3.7 Minocycline reduces astrocytic LDs and improves cognitive impairment in HH-exposed mice by inhibiting microglial inflammation.

Previous studies have demonstrated that minocycline effectively inhibits microglial activation [47,48]. In the current study, we found that minocycline treatment under hypoxic conditions significantly reduced neuroinflammation levels in the brain (Figure 7A,B, *p* < 0.05). To further investigate minocycline’s effect on astrocytic LD accumulation, we administered minocycline in a hypoxic co-culture system. The results showed that minocycline significantly attenuated hypoxia-induced LD accumulation in astrocytes (Figure 7C,D, *p* < 0.001). In animal experiments, minocycline similarly alleviated HH exposure-induced astrocytic LD accumulation and cellular overactivation (Figure 7E–I, *p* < 0.001). These findings indicate that minocycline, as an anti-inflammatory agent, effectively improves astrocytic LD accumulation by suppressing pro-inflammatory microglial activation and reducing neuroinflammatory responses.

**Figure 8:**
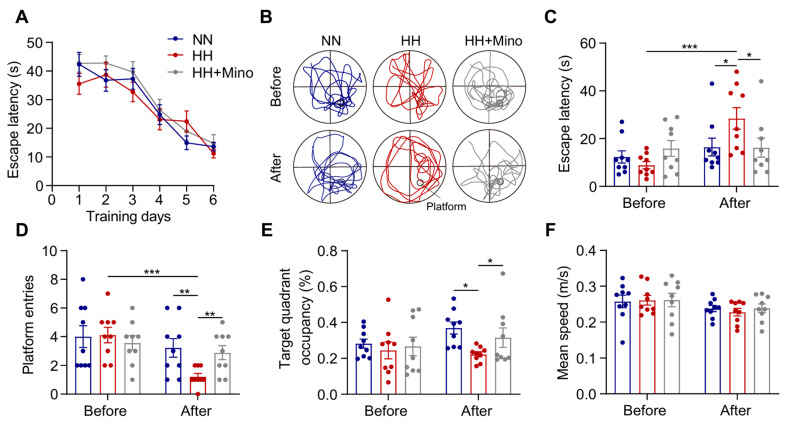
Minocycline alleviates HACI in mice. (**A**) Escape latency of C57BL/6 mice during the 6-day MWM training. (**B**) Swimming trajectories of mice before and after HH exposure (7000 m, 48 h) following minocycline treatment. (**C**) Latency to reach the target platform area in the probe test before and after HH exposure. (**D**) Number of crossings over the original platform area in the probe test. (**E**) Percentage of time spent in the target platform area during the probe test. (**F**) Average swimming speed of mice in the probe test. *n* = 9. Data were analyzed using two-way ANOVA followed by Sidak’s multiple comparisons test. * *p* < 0.05, ** *p* < 0.01 and *** *p* < 0.001. Blue line: NN; red line: HH, grey line: HH+Mino.

Based on these findings, we further evaluated minocycline’s protective effects against HACI. All mice completed MWM training prior to HH exposure and passed probe test validation, with no significant differences in baseline learning/memory capacity between groups (Figure 8A). When administered as a pretreatment before HH exposure, minocycline significantly ameliorated HH-induced memory impairment (Figure 8B). The minocycline-treated group showed significantly shorter latency in finding the hidden platform (Figure 8C, *p* < 0.05), increased platform crossings (Figure 8D, *p* < 0.01), and prolonged target quadrant dwell time (Figure 8E, *p* < 0.05) following HH exposure. Notably, minocycline had no significant effect on swimming speed (Figure 8F), excluding potential motor function interference with behavioral outcomes. These results demonstrate that minocycline improves hypoxia-induced cognitive dysfunction, highlighting its potential as a therapeutic candidate for high-altitude cerebral disorders (HACDs).

## 4. Discussion

Brain lipid homeostasis plays a pivotal role in maintaining cognitive function. This study is the first to reveal the involvement of disrupted brain lipid homeostasis in the pathogenesis of HACI. The key findings include the following: (1) significant accumulation of lipids, particularly cholesterol, was observed in the brain tissues of HACI mouse models, primarily stored as LDs in glial cells; (2) hypoxia initially induces LD accumulation in microglia and promotes their pro-inflammatory activation, leading to IL-1β release, which subsequently triggers LD accumulation in astrocytes; (3) microglia depletion markedly alleviates both hypoxia-induced and systemic inflammation-induced astrocytic LD accumulation and cognitive impairment; (4) minocycline effectively ameliorates hypoxia-induced glial LD accumulation and preserves cognitive function, demonstrating potential as a therapeutic candidate for preventing HACI onset and progression.

Brain diseases caused by lipid homeostasis imbalance have attracted widespread attention. Studies have shown that in mouse models of ischemic stroke, lipid droplet accumulation in microglia accompanied by upregulated neuroinflammation significantly exacerbates post-stroke brain injury progression [14]. In AD, aberrant lipid accumulation is observed in the upper granular layer of the frontal cortex, primarily characterized by elevated levels of phosphatidylcholine and sphingomyelin [49]. Additionally, chronic intermittent hypoxia induced by obstructive sleep apnea leads to massive LD accumulation in hippocampal neurons, microglia, and astrocytes, ultimately resulting in hippocampal neuronal death and cognitive impairment [50]. A high-glucose environment impairs LD autophagy in microglia, inducing LD accumulation and aggravating diabetes-associated cognitive impairment [44]. We are the first to demonstrate that cerebral lipid accumulation triggers HACI, further underscoring the critical role of brain lipid homeostasis in maintaining neuronal function. Notably, the deficiency of certain specific lipids also significantly impacts cognitive function. For instance, ω-3 polyunsaturated fatty acids play a pivotal role in childhood brain development, and their insufficiency triggers neuroinflammation [51], while a lack of medium-chain triglycerides accelerates cognitive decline in AD [52]. Recent studies indicate that astrocytic LD formation represents a double-edged sword in response to metabolic stress: on one hand, chronic LD accumulation may promote neuroinflammation; on the other hand, acute-phase LD formation likely serves as a crucial protective mechanism for detoxifying harmful lipids [29]. This duality underscores the necessity to evaluate the biological significance of LDs within specific pathological contexts [53]. Our findings reveal a selective accumulation of LDs in astrocytes but not in neurons following HH exposure (Figure S1), suggesting a potential neuron-to-astrocyte lipid redistribution mechanism under hypoxic stress [29]. This cell-type-specific response indicates that neurons may actively transfer excess fatty acids to neighboring astrocytes for storage and detoxification, revealing a novel aspect of intercellular metabolic coordination in response to hypoxia. Importantly, these observations demonstrate that HH exposure induces system-wide remodeling of cerebral lipid metabolism.

Alterations in lipid homeostasis may impair neuronal function by affecting key processes such as neuronal membrane fluidity, synaptic plasticity, and neurotransmitter release. For instance, imbalances in cholesterol and sphingomyelin can disrupt lipid raft structure, thereby interfering with the localization and function of NMDA receptors, impairing long-term potentiation, and ultimately leading to learning and memory deficits [54]. Additionally, the accumulation of certain lipid metabolites may promote oxidative stress and neuroinflammation, further exacerbating neuronal damage [55]. Cognitive functions in the prefrontal cortex might be compromised due to lipid-mediated disruptions in neurotransmitter signaling [56]. Furthermore, abnormal lipid metabolism may facilitate to sleep–wake behavior in Parkinson’s disease [57]. Thus, both excessive and insufficient lipid levels in the brain impair cognitive function. Therefore, drugs capable of stabilizing lipid homeostasis hold promising potential for cognitive protection.

As crucial immune effector cells in the CNS, microglia play a pivotal role in maintaining brain homeostasis. Our previous studies have demonstrated that abnormal microglial activation constitutes a key pathological mechanism in HACD [31]. Intriguingly, hypoxia does not directly damage astrocytes; rather, their observed swelling and aberrant activation are entirely dependent on the activation status of microglia [37,58]. The current study further reveals that hypoxia-induced cerebral lipid metabolic disturbances are also regulated by microglia—both pharmacological depletion of microglia and inhibition of their overactivation significantly ameliorate lipid homeostasis imbalance. These findings not only deepen our understanding of the central role of microglia and neuroinflammation in the pathogenesis of HACDs but also provide a theoretical foundation for anti-inflammatory therapies in preventing and treating altitude-related neurological diseases. Consequently, anti-inflammatory compounds such as catechins [59], Rhodiola rosea [60], and butylphthalide [61] may exhibit therapeutic potential in maintaining cerebral lipid homeostasis through the modulation of microglial function.

Minocycline is a semi-synthetic tetracycline antibiotic with oral bioavailability and blood–brain barrier permeability. Minocycline exhibits multiple pharmacological properties, including anti-cancer, anti-inflammatory, and glutamate antagonist effects [62,63]. Studies have demonstrated that minocycline significantly inhibits the pro-inflammatory activation of microglia [64,65]. Notably, minocycline ameliorates hypoxia-induced cerebral lipid homeostasis disruption by modulating microglial function. This finding implies that minocycline may have broad clinical applications in diseases associated with disrupted brain homeostasis, such as stroke, traumatic brain injury, and AD. Furthermore, given the homology between microglia and macrophages, minocycline may also exert therapeutic effects in macrophage-mediated pathologies, including atherosclerosis, glioblastoma, and hepatocellular carcinoma [66,67,68]. Therefore, the potential therapeutic value of minocycline across various diseases warrants further investigation.

Recent studies have increasingly demonstrated that minocycline exhibits typical hormesis effects, whereby low doses exert neuroprotective effects by inhibiting signaling pathways such as TLR4/NF-κB, while higher doses may produce opposite effects. This characteristic provides new insights for understanding the complex role of minocycline in neurological disorders and supports the rationale for selecting low-dose regimens in our study. Indeed, the hormesis phenomenon has been observed in various neuroprotective agents [69,70], suggesting that moderate stress stimulation may activate endogenous protective mechanisms, which holds significant implications for developing novel neuroprotective strategies.

This study has several limitations: First, the high-altitude exposure model only simulated HH conditions without incorporating other environmental factors such as radiation and low temperature, which may synergistically affect cognitive function. Second, this study only examined cognitive function during an 8-day recovery period at low altitude, lacking longer-term follow-up data to assess sustained recovery effects. Third, while the research focused on changes in microglia, astrocytes, and neurons, it failed to investigate the impact of high-altitude exposure on lipid homeostasis in other neural cell types. Fourth, although low-dose minocycline showed therapeutic potential, this study did not evaluate its long-term biosafety profile, limiting its clinical applicability. Finally, by inhibiting the CSF1R signaling pathway, PLX5622 not only depletes microglia in the central nervous system but also reduces macrophages in peripheral tissues, such as the spleen and bone marrow. This systemic depletion may impair immune surveillance and disrupt hematopoietic homeostasis [71]. Furthermore, the decline in intestinal macrophage populations could perturb gut microbiota balance, potentially modulating neuroinflammatory processes via the microbiota–gut–brain axis [72]. Thus, when employing PLX5622 to investigate the role of microglia in cerebral lipid homeostasis, the potential confounding effects of peripheral macrophage depletion must be carefully considered.

In this study, we observed abnormal cholesterol accumulation in the brain. Recent studies have demonstrated that cholesterol homeostasis, particularly impaired cholesterol turnover, plays a pivotal role in inducing cognitive impairment [73,74,75]. However, it remains unclear whether the LD accumulation in glial cells of HACI mice results from increased cholesterol levels. Furthermore, the mechanism underlying hypoxia-induced lipid accumulation in glia cells at high altitudes warrants further investigation, as this represents a crucial clinical need for preventing and treating HACI. Our finding that IL-1β induces LD accumulation in astrocytes indirectly supports the hypothesis that systemic inflammation triggers HACDs [76,77]. Notably, activated microglia release not only IL-1β but also other inflammatory factors, including IL-6, TNF-α, and IL-18—their potential involvement in disrupting astrocytic lipid homeostasis merits additional exploration. Collectively, this study is the first to establish the significant role of cerebral lipid homeostasis in HACI and identifies microglia-mediated immune dysregulation as a key contributor to cerebral lipid metabolic disturbances. While these findings are preliminary and numerous questions remain unresolved, they suggest that metabolic alterations in the central nervous system may represent a novel research avenue for understanding HACDs, offering fresh perspectives for both mechanistic studies and clinical interventions.

## 5. Conclusions

This study demonstrates that disrupted brain lipid homeostasis, characterized by significant LD accumulation, plays a critical role in the pathogenesis of HACI. Key findings reveal that hypoxia induces LD accumulation in microglia, driving their pro-inflammatory activation and IL-1β release, which subsequently promotes lipid deposition in astrocytes. Microglia depletion significantly mitigates both hypoxia- and systemic inflammation-induced astrocytic lipid accumulation and cognitive impairment. Notably, minocycline effectively alleviates glial lipid droplet accumulation and preserves cognitive function, highlighting its potential as a therapeutic intervention for preventing HACI onset and progression.

## Data Availability

The authors confirm that the data supporting the findings of this study are available within the article and its Supplementary Material. Raw data that support the findings of this study are available from the corresponding author, upon reasonable request.

## Supplementary Materials

The following supporting information can be downloaded at: https://www.mdpi.com/article/10.3390/antiox14080918/s1, Figure S1: HH exposure does not significantly affect LDs in neurons of the CA1 region in mice.

## Abbreviations

AD, Alzheimer’s disease; CNS, central nervous system; CSF1R, colony-stimulating factor 1 receptor; HACD, high-altitude cerebral disorders; HACI, high-altitude cognitive impairment; HCM, hypoxic conditional medium; HH, hypobaric hypoxia; LD, lipid droplet; LPS, lipopolysaccharide; MOR, Morris water maze; NCM, normoxic conditioned medium; Nig, nigericin; NN, normobaric normoxia; NOR, Novel object recognition; PLIN2, Perilipin2.
