# Supplementary material for: Pro-Inflammatory Microglia Exacerbate High-Altitude-Induced Cognitive Impairment by Driving Lipid Droplet Accumulation in Astrocytes
Source: Antioxidants (Basel). 2025 Jul 26;14(8):918. doi: 10.3390/antiox14080918 (PMC12383157; doi:10.3390/antiox14080918)
Supplement: Supplementary file 1 [file antioxidants-14-00918-s001.zip › antioxidants-3701903-supplementary.pdf]

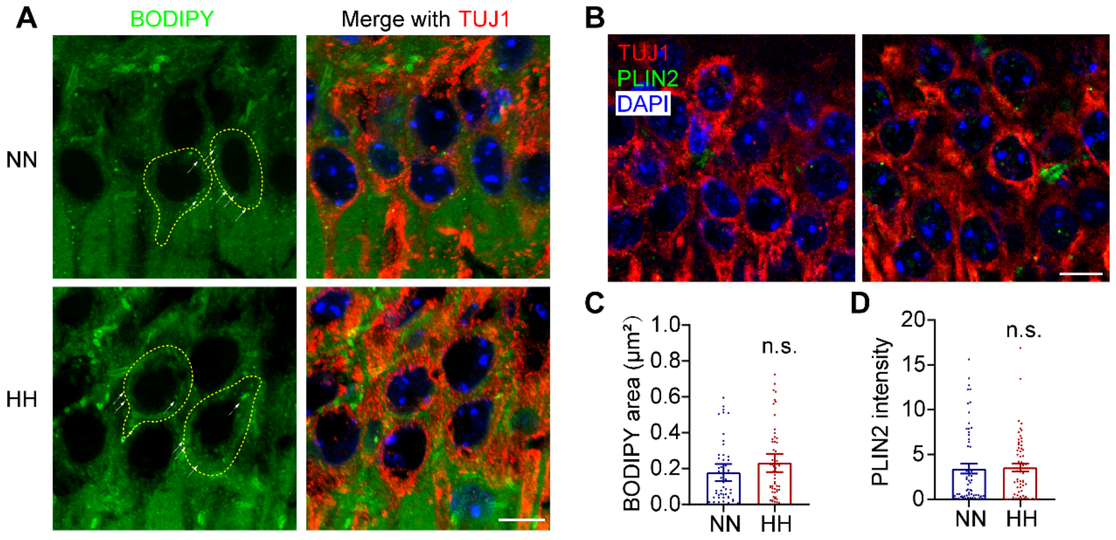

Supplementary Figure 1. HH exposure does not significantly affect LDs in neurons of the CA1 region in mice. (**A**) Representative images of mouse brain sections immunolabeled with anti-TUJ1 antibody and co-stained with BODIPY 493. (**B**) Representative images of mouse brain sections immunolabeled with anti-TUJ1 and anti-PLIN2 antibodies and counterstained with DAPI. (**C**) Quantitative analysis of the BODIPY 493 fluorescence area in $TUJ1^+$ cells in the CA1 region (n = 60; 12 cells per mouse from 5 mice per group). (**D**) Quantitative analysis of PLIN2 fluorescence intensity in $TUJ1^+$ cells in the CA1 region (n = 60; 12 cells per mouse from 5 mice per group). Scale bar in fluorescence images = 10 μm. Data were analyzed using Student's *t*-test. n.s., not significant.
